# 1,6-Hexanediol, commonly used to dissolve liquid–liquid phase separated condensates, directly impairs kinase and phosphatase activities

**DOI:** 10.1016/j.jbc.2021.100260

**Published:** 2021-01-08

**Authors:** Robert Düster, Ines H. Kaltheuner, Maximilian Schmitz, Matthias Geyer

**Affiliations:** The Institute of Structural Biology, University of Bonn, Bonn, Germany

**Keywords:** 1,6-Hexanediol, Phase separation, LLPS, CDK7, CDK9, P-TEFb, RNA polymerase II CTD, Cdk, Cyclin-dependent kinase, CTD, C-terminal domain (of RNA polymerase II), DYRK1A, dual-specificity tyrosine phosphorylation regulated kinase 1A, GSH, glutathione, GST, glutathione S-transferase, IDR, intrinsically disordered region, LLPS, liquid–liquid phase separation, nanoDSF, nano differential scanning fluorimetry, P-TEFb, positive transcription elongation factor b, PBS, phosphate-buffered saline, TFIIH, transcription factor II human

## Abstract

The concept of liquid–liquid phase separation (LLPS) has emerged as an intriguing mechanism for the organization of membraneless compartments in cells. The alcohol 1,6-hexanediol is widely used as a control to dissolve LLPS assemblies in phase separation studies in diverse fields. However, little is known about potential side effects of 1,6-hexanediol, which could compromise data interpretation and mislead the scientific debate. To examine this issue, we analyzed the effect of 1,6-hexanediol on the activities of various enzymes *in vitro*. Already at 1% volume concentration, 1,6-hexanediol strongly impaired kinases and phosphatases and partly blocked DNA polymerases, while it had no effect on DNase activity. At concentrations that are usually used to dissolve LLPS droplets (5–10%), both kinases and phosphatases were virtually inactive. Given the widespread function of protein phosphorylation in cells, our data argue for a careful review of 1,6-hexanediol in phase separation studies.

The formation of membraneless organelles by liquid–liquid phase separation (LLPS) is a fast-growing biological concept, bringing new insights into a multitude of cellular processes such as signal transduction, gene expression, stress response, and the regulation of transcription (1, 2, 3, 4, 5). Different nuclear assemblies (the nucleolus, nuclear speckles, Cajal bodies, and the nuclear pore), as well as cytoplasmic structures (P-bodies, stress granules, and the centriole) form without defining membranes by the condensation of proteins, nucleic acids, and other biomolecular components (6, 7). Moreover, accumulating evidence points to the implication of LLPS in aggregate formation of pathological proteins, including FUS accumulation in amyotrophic lateral sclerosis (ALS), amyloid- and tauopathies in neurodegenerative diseases, but also viral infections and cancer (8, 9, 10, 11, 12, 13, 14, 15, 16). A recent study indicates that even drug pharmacodynamics of small-molecular cancer therapeutics are altered by the concentration of compounds in specific protein condensates independent of their drug targets, resulting in a change of drug activity (17). These results implicate that a better understanding of LLPS might lead to substantial advantages in the future development of efficacious therapeutics in many diseases.

A hallmark of many proteins, which are prone to participate in LLPS assemblies, is the presence of intrinsically disordered regions (IDRs). These regions are enriched in repetitive sequences of a few amino acids, usually resulting in characteristic domains of low complexity (18, 19). A growing number of studies identified and characterized IDRs of various LLPS participating proteins *in vitro* and in cells, demonstrating that IDRs play a crucial role in liquid droplet formation of these proteins under certain conditions. Previous studies observed the formation of such condensates also for the C-terminal domain (CTD) of human RNA polymerase II (pol II) and other proteins involved in transcriptional control (20, 21). The human RNA pol II CTD is composed of 52 hepta-peptide repeats with a YSPTSPS consensus sequence, provoking a structure of low complexity and a plethora of amino acids, which can be targeted by posttranslational modifications (22). During transcription, the pol II CTD is phosphorylated in a specific manner upon the transitions from initiation to elongation, RNA procession, polyadenylation, and termination. Whereas the hypo-phosphorylated pol II CTD is incorporated into Mediator condensates during transcription initiation, this incorporation is reduced upon phosphorylation by the TFIIH kinase Cdk7 and the hyperphosphorylated pol II CTD is subsequently incorporated into condensates that are formed by splicing factors (23). Thus, pol II CTD phosphorylation might be a persuasive mechanism that is suggested to regulate the condensate preferences of RNA pol II during transcription. The stability of such pol II CTD condensates was shown to decrease upon truncation to 25 hepta-repeats, whereas extension to 70 hepta-repeats had the opposite effect (21). Likewise, P-TEFb and DYRK1A, two key players in transcriptional regulation and well-studied pol II CTD kinases, have recently been shown to participate in liquid-like droplets *via* a histidine-rich domain of low complexity (24).

The aliphatic alcohol 1,6-hexanediol interferes with weak hydrophobic interactions and is often used to dissolve protein condensates *in vitro* and in cells, illustrating the reversible character of phase separations (25, 26). The first report of an aliphatic dialcohol used to disturb membraneless subcellular structures was a study on nuclear pore complexes where 1,2-hexanediol and the cyclic variant cyclohexane-1,2-diol were capable to perturb the permeability barrier of the nuclear pore, whereas the less hydrophobic 1,2,3-hexanetriol had no effect (27). Another study on nuclear transport mechanisms tested different alcohols and introduced 1,6-hexanediol as a potent agent to induce permeability of the nuclear pore and showed that the potency of the alcohol to dissolve condensates is correlated to its hydrophobicity (28). Since then, the vulnerability of condensates to 1,6-hexanediol treatment is often used to delineate that droplet structures are caused by phase separation. However, LLPS is driven by different types of interactions including electrostatic, hydrophobic, pi–pi, and pi–cation interactions (15, 29). It is not yet fully understood how 1,6-hexanediol disrupts these assemblies and several protein condensates are indeed resistant to 1,6-hexanediol treatment *in vitro* (8, 25). Moreover, 1,6-hexanediol treatment of yeast and human cells revealed hexanediol resistant spots in cells as well as the appearance of such structures upon treatment (30, 31). Albeit tolerated at concentrations of up to 10% for short times, prolonged incubation with 1,6-hexanediol results in cell death (3, 31). Besides affecting the nuclear pore complex, hexanediol has been shown to dissolve intermediate filaments, which could contribute to the cellular phenotypes of the chemical (32). Notably, 1,6-hexanediol has been suggested as a specific inhibitor of lactate dehydrogenase isoenzyme 1 already in the late 80s of the last century (33, 34). The capacity of 1,6-hexanediol as a marker to distinguish LLPS from other assemblies is therefore limited, in particular in cellular systems in which the experimental conditions cannot be controlled adequately.

In addition to the mentioned caveats of 1,6-hexanediol, direct effects of the dialcohol on protein function and integrity besides its ability to dissolve condensates have not been systematically addressed (26, 30). In this study, we investigated the effect of 1,6-hexanediol on the enzymatic activities of kinases and phosphatases in *in vitro* assays. Our data suggest that 1,6-hexanediol impairs kinase activity kinome-wide independently of LLPS. Hexanediol is therefore not a suitable tool to study the functional relationship between phase separation and cellular pathways that involve phosphorylation. We conclude that studies using 1,6-hexanediol should be very carefully interpreted with regard to mechanistic reasoning from experiments using this agent.

## Results

### 1,6-Hexanediol impairs kinase activity irrespectively of LLPS formation

**Figure 1:**
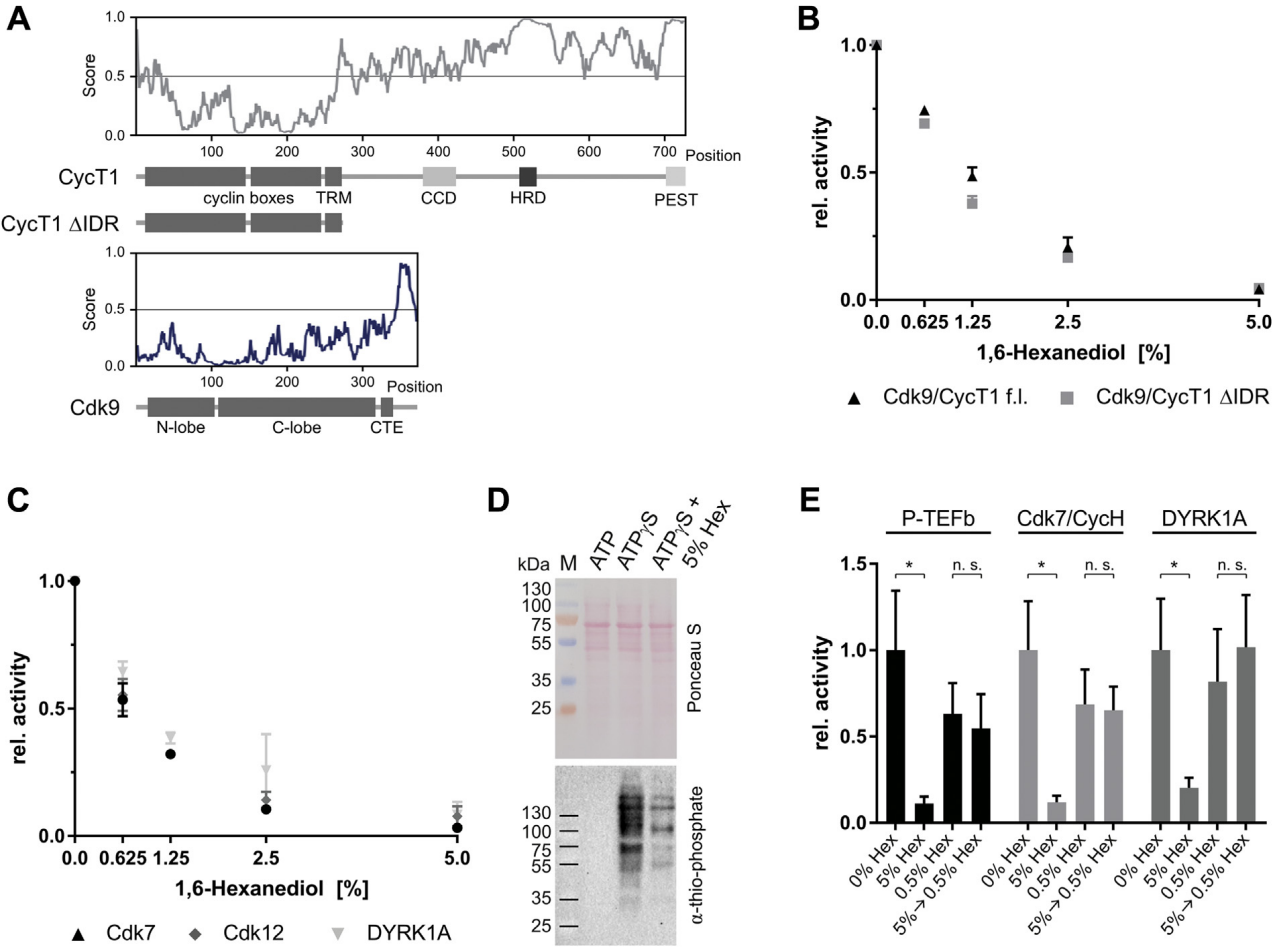
**1,6-Hexanediol impairs kinase activity in a dose-dependent manner.***A*, prediction of unstructured regions within CycT1 (*gray line*) and Cdk9 (*blue line*) using the program IUPred2A (57). Below is a schematic representation of the P-TEFb protein constructs used in this study. The domain architecture indicates the cyclin boxes, Tat-recognition motif, coiled-coil domain, histidine-rich domain and PEST sequence of CycT1, and the N- and C-terminal lobe and the C-terminal extension sequence of Cdk9. *B*, kinase activity assay in the presence of increasing concentrations of 1,6-hexanediol. Kinase activity was analyzed in a radiometric assay by liquid scintillation counting using [^32^P]-γ-ATP. Kinase assays contained 0.2 μM Cdk9 complex, 10 μM GST-CTD_[52]_, 1 mM ATP, and indicated concentrations (v/v) of 1,6-hexanediol. Reactions were terminated after incubation for 15 min at 30 °C by addition of EDTA. The activity of the kinases is shown as relative values normalized to the absence of 1,6-hexanediol. *C*, same as in B, but for transcription kinases Cdk7/CycH, Cdk12/CycK, and DYRK1A. *D*, kinase assay using HeLa full cell lysate. The lysate was incubated for 1 h at 30 °C with MgCl_2_ and ATP or ATPγS with and w/o 5% 1,6-hexanediol. Ponceau S staining illustrates the equal loading of the lanes (*upper panel*). The thio-phosphorylation was detected with a specific antibody by western blotting indicating a strong decrease over all HeLa cell kinases upon addition of hexanediol (*lower panel*). *E*, Kinase activity recovers after dilution from 5% to 0.5% 1,6-hexanediol. Transcription kinases Cdk7/CycH, P-TEFb, and DYRK1A were incubated at 10x kinase concentration in 5% 1,6-hexanediol for 30 min at room temperature and subsequently diluted to 0.5% of the alcohol. All kinases regained the activity upon dilution to 0.5% 1,6-hexanediol to comparable levels of freshly incubated enzymes, suggesting that the alcohol is reversibly washed out from the proteins. Samples were performed in triplicates and analyzed for significant differences using two-sided, nonpaired t-test. ∗*p*-value < 0.05, n.s., not significant.

We started the analysis of 1,6-hexanediol on kinase activity with the cyclin-dependent kinase (Cdk) complex Cdk9/CycT1, which acts as the positive transcription elongation factor b (P-TEFb) by phosphorylating the RNA pol II CTD and other regulators of transcriptional elongation. P-TEFb has been found to form condensates due to an intrinsically disordered region within the CycT1 subunit (Fig. 1*A*). Furthermore, LLPS formation has been suggested to facilitate Cdk9 activity since 1,6-hexanediol treatment greatly diminished P-TEFb activity *in vitro* (24).

To analyze the impact of 1,6-hexanediol, we purified full-length P-TEFb from *Sf9* cells and determined its ability to phosphorylate the C-terminal domain (CTD) of the human RNA pol II subunit Rpb1 in the absence and presence of the dialcohol. We additionally used a truncated P-TEFb variant (P-TEFb ΔIDR), which lacks the disordered C-terminal region of CycT1 and is thus unable to form condensates (24). Addition of 1,6-hexanediol results in a concentration-dependent decrease in kinase activity in both full-length P-TEFb and the truncated P-TEFb ΔIDR (Fig. 1*B*). Kinase activity was already diminished by 25% at a 1,6-hexanediol concentration of 0.625% (v/v). At a concentration of 5% 1,6-hexanediol, the kinase activity was reduced to 5%. Importantly, the effect was independent of the CycT1 disordered region suggesting that impaired kinase activity by 1,6-hexanediol is not related to LLPS.

To further investigate the effect of 1,6-hexanediol on kinases, we expanded our *in vitro* analysis to the human transcription kinases Cdk7/CycH, Cdk12/CycK, and DYRK1A (Fig. 1*C*). In this context, we only used well-described, structured regions of the proteins, which have not been implicated to form condensates *in vitro*. Moreover, kinases were used at a concentration of 0.1 μM and without any crowding agents, which makes it unlikely that they phase-separated in the tube. All three kinases were similarly affected by the use of 1,6-hexanediol as observed before for P-TEFb, demonstrating that 1,6-hexanediol impairs kinase activity independently of their capability for LLPS.

To overcome a technical bias due to the use of recombinant proteins, we further analyzed native kinases and substrates from HeLa full cell lysate. To monitor kinase activity in the samples and to discriminate *in vitro* phosphorylation from already phosphorylated proteins, we used ATPγS in combination with a specific antibody, which detects the thio-phosphorylation after alkylation with p-nitrobenzyl mesylate (35). After cell lysis, we added MgCl_2_ and either ATP or ATPγS and incubated the samples for 1 h at 25 °C in the absence or presence of 5% 1,6-hexanediol. Equal loading of the samples was confirmed by ponceau staining (Fig. 1*D*). Western blot analysis showed no signal after incubation of HeLa lysate with ATP, assuring the specificity of the antibody for thio-phosphorylated proteins. Incubation with ATPγS led to pronounced band patterns showing efficient use of ATPγS by the native kinases. Consistent with our data obtained from recombinant proteins, the addition of 5% 1,6-hexanediol greatly diminished kinase activity within the HeLa cell lysate suggesting a general, kinome-wide effect of 1,6-hexanediol on kinase activity.

We next wondered whether the effect of 1,6-hexanediol on kinase activity reduction *in vitro* is reversible. Therefore, we preincubated 1 μM recombinant kinases P-TEFb, Cdk7/CycH, or DYRK1A with 5% 1,6-hexanediol for 30 min at room temperature. These samples were then diluted tenfold to 0.5% 1,6-hexanediol by addition of kinase assay buffer. For comparison, fresh kinase samples at 0.1 μM concentration without or with 0.5% 1,6-hexanediol were prepared. As expected, addition of 5% 1,6-hexanediol significantly diminishes the activity of all three kinases (Fig. 1*E*). However, upon dilution of these samples from 5% to 0.5% 1,6-hexanediol, the kinases regained activity to the same levels as freshly prepared kinases with 0.5% 1,6-hexanediol. We therefore conclude that the alcohol 1,6-hexanediol does not irreversibly impair the kinase activity but can be washed out upon dilution from higher concentrates to restore the protein’s enzymatic activity.

### 1,6-Hexanediol affects phosphatase and polymerase but not DNase activity

**Figure 2:**
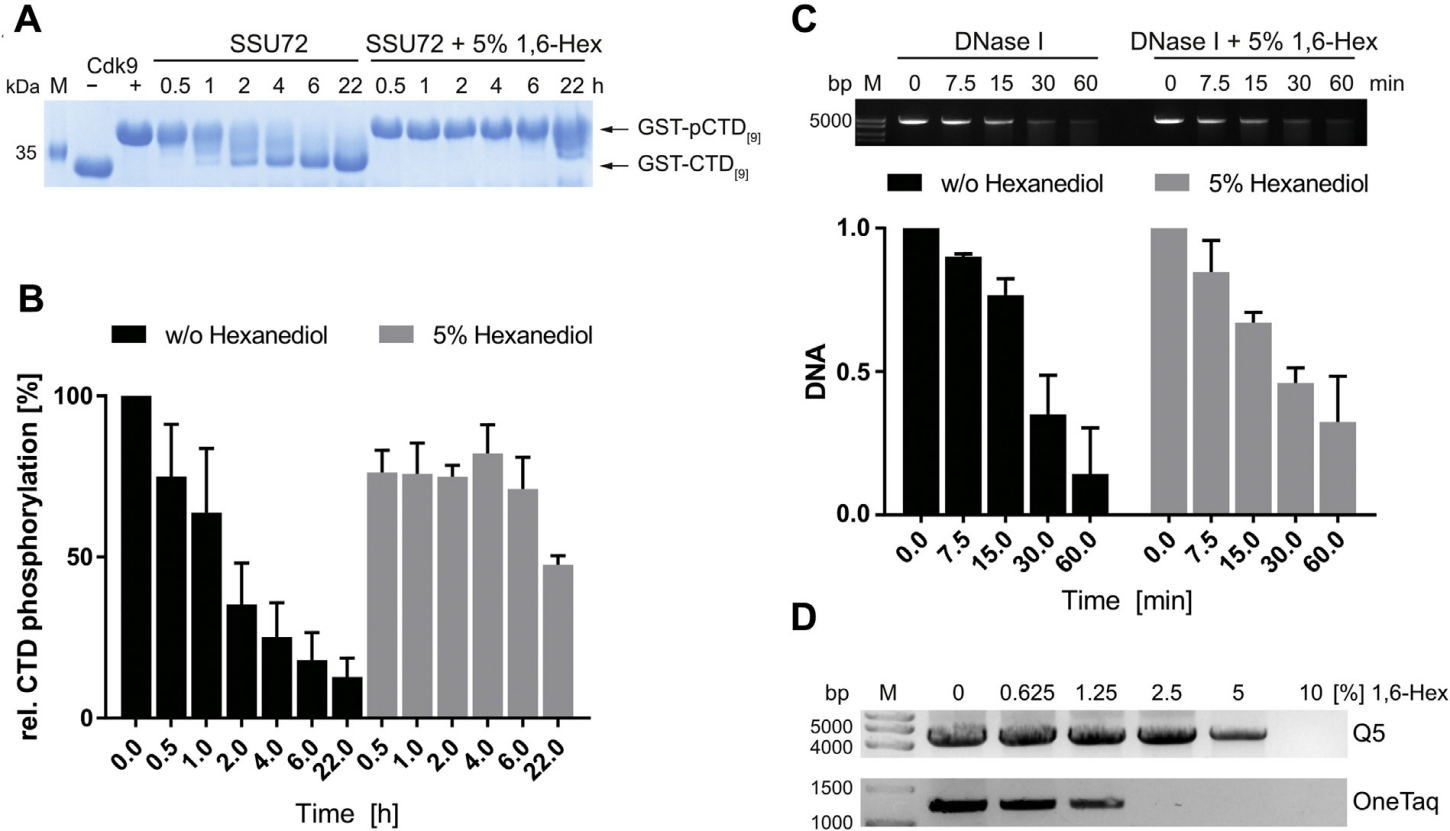
**1,6-Hexanediol affects phosphatase and polymerase but not DNase activity.***A*, P-TEFb phosphorylated GST-CTD_[9]_ was dephosphorylated by SSU72 in the absence and presence of 5% 1,6-hexanediol. Samples were taken at indicated time points and migration behavior was visualized by Coomassie-stained SDS-PAGE analysis. *B*, quantitative analysis of CTD dephosphorylation by the phosphatase SSU72 with and w/o 5% 1,6-hexanediol in time course experiments. For quantification, samples were phosphorylated with radioactive [^32^P]-γ-ATP and analyzed by liquid scintillation counting. Data are depicted as mean ± SD. *C*, linearized plasmid DNA was digested with DNase I at 37 °C in the absence or presence of 1,6-hexanediol. The DNA was visualized by peqGreen staining using a BioRad XRS+ gel documentation system (*upper panel*). DNA was quantified from three replicates and normalized to the respective 0 min sample (*lower panel*). Data are depicted as mean ± SD. *D*, PCR amplification assays with DNA polymerases Q5 (*top*) or OneTaq (*bottom*) upon increasing concentrations of 1,6-hexanediol from 0 to 10%. PCR products were subjected to 1% agarose gels. One representative experiment out of three is shown for each polymerase.

In cells, the function of kinases on substrate phosphorylation is counteracted by phosphatases. The strong effect of 1,6-hexanediol towards kinase activity prompted us to investigate if phosphatases are similarly affected. We expressed and purified the RNA pol II CTD specific phosphatase SSU72 from *E.coli* and determined its ability to dephosphorylate P-TEFb phosphorylated GST-CTD composed of nine consensus pol II CTD repeats (GST-CTD_[9]_) by SDS-PAGE and quantitative radiometric assays (Fig. 2, *A* and *B*). The GST-CTD_[9]_ substrate was phosphorylated with 0.2 μM P-TEFb for 1 h at 30 °C resulting in a complete shift of the GST-CTD substrate in SDS-PAGE analysis indicating full phosphorylation of the substrate. P-TEFb activity was then quenched by addition of the kinase inhibitor Flavopiridol to a final concentration of 1 μM. Incubation of phosphorylated GST-CTD_[9]_ (GST-pCTD_[9]_) with the phosphatase SSU72 led to a continuous dephosphorylation of GST-CTD_[9]_ (Fig. 2*A*). However, in the presence of 5% 1,6-hexanediol, phosphatase activity was nearly absent and only visible after 22 h of incubation (Fig. 2*A*). For quantification we repeated the assay with isotope-labeled [^32^P]-γ-ATP and subsequent detection by liquid scintillation counting (Fig. 2*B*). Consistent with SDS-PAGE analysis, incubation of phosphorylated GST-CTD_[9]_ with the phosphatase SSU72 resulted in a constant decrease in phosphorylation levels. In the presence of 5% 1,6-hexanediol, dephosphorylation was reduced and only detectable after 22 h of incubation.

We wondered if other enzymatic activities are similarly affected by 1,6-hexanediol and chose DNA digestion by DNase I and amplification by DNA polymerases as complementary systems to the kinase and phosphatase assays. Linearized plasmid DNA was digested with DNase I in the absence or presence of 5% 1,6-hexanediol over a time course of 60 min (Fig. 2*C*). Without 1,6-hexanediol treatment, the plasmid DNA was digested almost completely after 60 min. However, in contrast to the observed effects on kinases and phosphatases activity, the ability of DNase I to digest plasmid DNA was not significantly affected by the presence of 5% 1,6-hexanediol as the DNA was similarly degraded over the time course experiment.

The effect of 1,6-hexanediol on DNA amplification activities using polymerases Q5 and OneTaq in a PCR assay instead varied. Whereas the Q5 high-fidelity polymerase using a DNA template with high GC content was only significantly reduced at 5% hexanediol concentration and fully impaired at 10%, the OneTaq polymerase appeared more susceptible to 1,6-hexanediol showing a reduced activity already at the lowest concentration applied (Fig. 2*D*). Here, the PCR activity was already diminished at 0.625% and fully lost at 2.5% of the dialcohol. The data nicely confirm that each enzyme reacts differently on the chemical and that the activity should be determined individually when interpreting dissolution experiments.

### 1,6-Hexanediol destabilizes proteins but does not impair their overall structure

**Figure 3:**
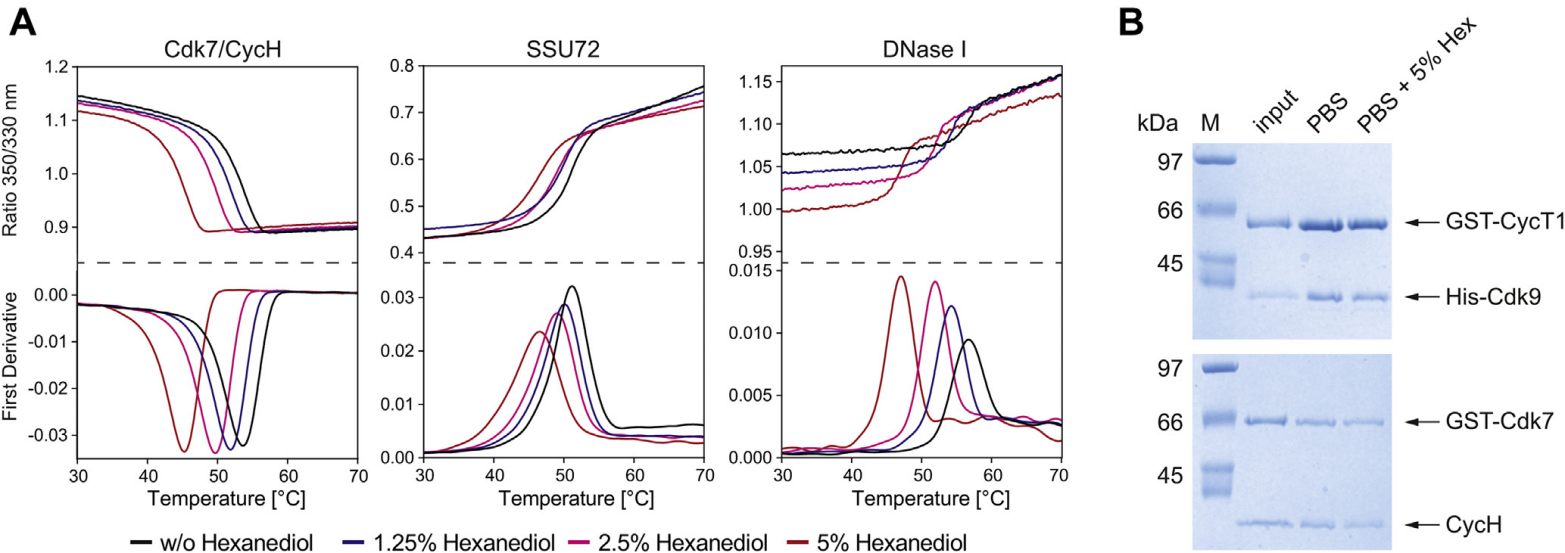
**1,6-Hexanediol destabilizes proteins but does not impair their overall integrity at concentrations up to 5%.***A*, thermal stability measurements of Cdk7/CycH, SSU72, and DNase I by nanoDSF. Protein unfolding is monitored by a change in the fluorescence ratio at 350/330 nm upon heating. The peak of the first derivative of the measurement delineates the melting point of the protein, which decreases continuously upon increasing concentrations of 1,6-hexanediol. *B*, pull-down of GST-CycT1-ΔIDR/Cdk9 and GST-Cdk7/CycH complexes. CDK-Cyclin complexes were coupled to glutathione 4B sepharose beads and incubated with PBS or PBS plus 5% 1,6-hexanediol. After washing, proteins were eluted in SDS-sample buffer and subjected to SDS-PAGE analysis. Input shows the protein prior to incubation.

We reasoned that the effect of 1,6-hexanediol is due to changes in the three-dimensional protein structure or its potential surface coverage. We used the nano differential scanning fluorimetry (nanoDSF) technique to determine how 1,6-hexanediol affects the thermal denaturation of the kinase Cdk7/CycH, the phosphatase SSU72, and the endonuclease DNase I. NanoDSF utilizes intrinsic tryptophan and tyrosine fluorescence to monitor protein unfolding. Thermal stability nanoDSF measurements showed that 1,6-hexanediol destabilized all tested proteins in a concentration dependent manner (Fig. 3*A*). Cdk7/CycH exhibited thermal denaturation at 53.4 °C, while the addition of 5% 1,6-hexanediol destabilized Cdk7/CycH to 45 °C. Thermal stability of SSU72 phosphatase was reduced upon addition of 1,6-hexanediol by 4.6 °C from 51.1 to 46.5 °C, whereas DNase I displayed an inflection point of denaturation at 56.7 °C, which was lowered to 47 °C upon addition of 5% 1,6-hexanediol.

For the examination of proper protein folding and accessibility, pull-down experiments with Cdk9/GST-CycT1 (1–272) and GST-Cdk7/CycH were used to clarify whether the overall complex formation of CDKs and cyclins was affected by 1,6-hexanediol. Cdk9/GST-CycT1 (1–272) and GST-Cdk7/CycH were coupled to GSH sepharose beads and incubated in PBS or in PBS containing 5% 1,6-hexanediol for 1 h (Fig. 3*B*). Beads were collected by centrifugation and washed three times in PBS or PBS-hexanediol. In contrast to the pronounced effects on protein stability, addition of 5% 1,6-hexanediol did not affect the Cdk/Cyclin interaction in pull-down assays. Neither the overall binding of GST to the GSH sepharose beads nor the Cdk/Cyclin interaction was affected, indicating that the overall structure of the proteins was not impaired at 5% 1,6-hexanediol.

### Dissolution of RNA pol II CTD condensates requires high concentration of 1,6-hexanediol

**Figure 4:**
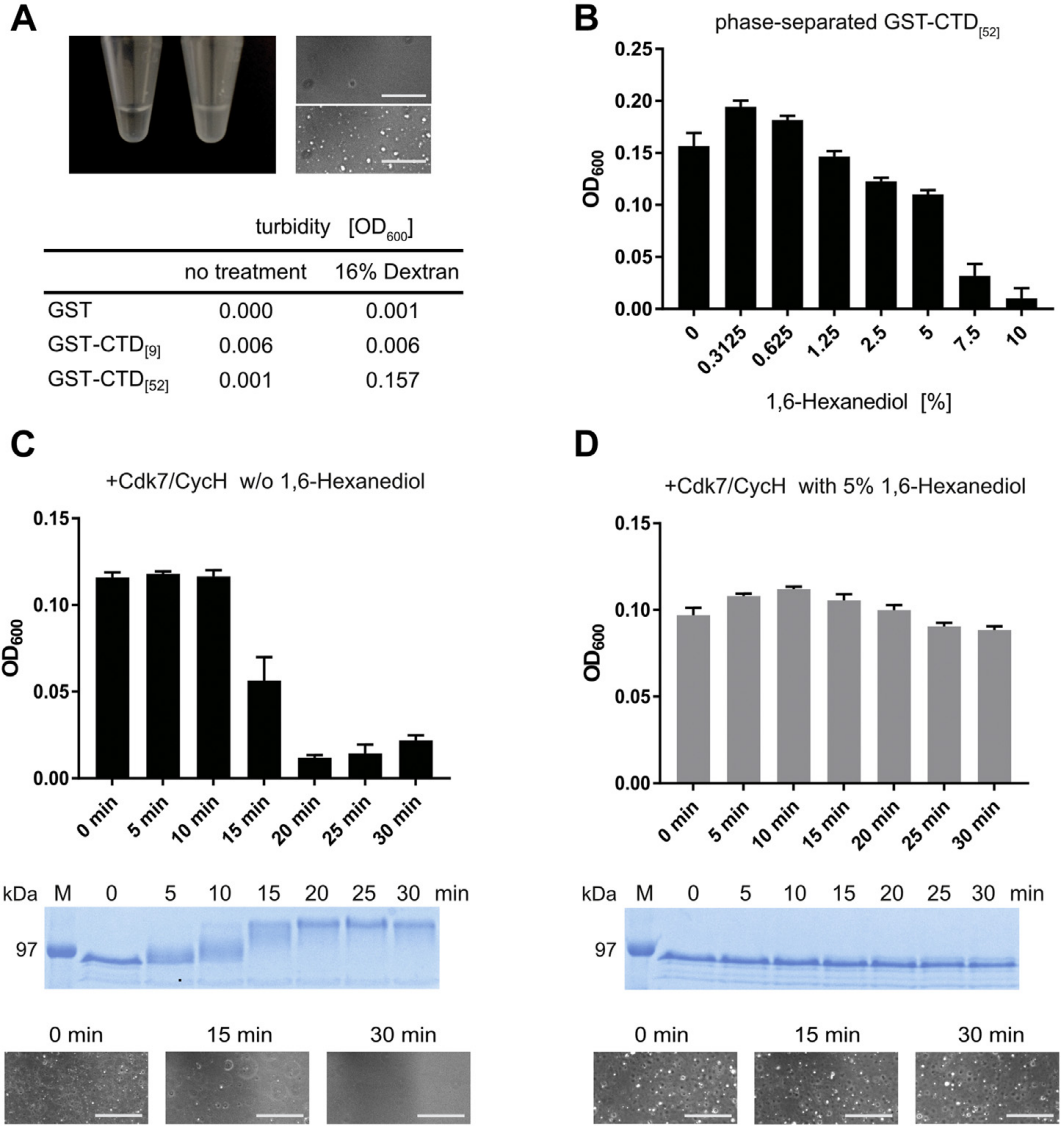
**Dissolution of RNA pol II CTD condensates requires high concentration of 1,6-hexanediol rendering Cdk7 inactive.***A*, solution of 10 μM full-length human GST-CTD_[52]_ in aqueous buffer (*left*). After addition of dextran, the GST-CTD_[52]_ sample turns turbid (middle). Microscopic images confirm the formation of liquid droplets (right images, top w/o dextran and bottom with dextran). The turbidity of 10 μM GST-CTD_[52]_ protein samples containing 9 or 52 hepta-repeats before and after addition of dextran was determined at 600 nm (OD_600_). Scale bars, 25 μm. *B*, OD_600_ measurements of phase-separated GST-CTD_[52]_ upon increasing concentrations of 1,6-hexanediol. Data represent mean ± SD from three replicates. *C*, dissolution of GST-CTD_[52]_ droplets by phosphorylation with Cdk7/CycH. In total, 10 μM GST-CTD_[52]_ was incubated with 0.1 μM Cdk7/CycH and 2 mM ATP in kinase assay buffer containing 16% dextran. The turbidity of the samples was determined at indicated time points in duplicate measurements. For analysis of the phosphorylation status, the reaction was quenched by mixing with 2× SDS sample buffer and subsequent analysis of 2 μg GST-CTD_[52]_ in a 12% SDS-PAGE. Microscopic images at three time points revealing the dissolution of condensates are shown at the bottom. Scale bars, 25 μm. *D*, at 5% 1,6-hexanediol GST-CTD_[52]_ droplets remain intact, whereas the kinase activity of Cdk7/CycH is impaired. The measurement was performed similarly as in *C* but in the presence of 5% 1,6-hexanediol. The stability of the condensates is confirmed by light microscopy images (bottom). Scale bars, 25 μm.

In a previous study, a CTD construct comprising all 52 repeats of the human RNA pol II was shown to undergo LLPS in the presence of 16% dextran *in vitro*, which can be monitored in a photometer by an increase in turbidity at a wavelength of 600 nm and by light microscopy (21). We were interested whether pol II CTD condensates could be dissolved at low 1,6-hexanediol concentrations, at which kinase activity is only mildly affected. For turbidity measurements, we incubated 10 μM human GST-CTD_[52]_ in PBS with 16% dextran and increasing concentrations of 1,6-hexanediol at room temperature. The GST-CTD_[52]_ sample turned turbid upon addition of dextran resulting in a change of the OD_600_ from 0.001 to 0.157 (Fig. 4*A*). Concomitantly, neither 16% dextran alone, nor a GST control, nor the truncated GST-CTD_[9]_ showed a change in turbidity at 16% dextran. The formation of droplets was confirmed by light microscopy. In the next experiment, we first formed phase-separated GST-CTD_[52]_ condensates upon incubation with 16% dextran. When incubated afterward with 1,6-hexanediol, low concentrations were not able to dissolve the GST-CTD_[52]_ condensates (Fig. 4*B*). In fact, OD_600_ values were even slightly increased in the presence of 0.3125% and 0.625% hexanediol. Interestingly, the dissolution of condensates does not seem to follow a linear or exponential decline, but rather required a specific, minimal concentration. A marked reduction in turbidity was only observed at hexanediol concentrations of 7.5% and 10%. These data reveal that the dissolution of GST-CTD_[52]_ condensates requires high concentrations of 1,6-hexanediol at which kinases and phosphatases become virtually inactive.

We wondered whether the impairment in kinase activity by 1,6-hexanediol persists in the presence of phase-separated pol II CTD. Previous experiments have shown that pol II CTD droplets can be resolved upon phosphorylation by Cdk7 (21). We reasoned that the presence of 1,6-hexanediol would significantly slow down or even inhibit this process by impairing kinase activity. We therefore monitored pol II CTD droplets photometrically during incubation with Cdk7/CycH and ATP (Fig. 4*C*, upper panel). Subsequently, we visualized the phosphorylation status of the GST-CTD by SDS-PAGE analysis showing a shift in migration from the unphosphorylated CTD_[52]_ IIa form to the hyperphosphorylated IIo form (Fig. 4*C*, lower panel). Incubation of phase-separated pol II CTD with Cdk7 dissolves the droplets within 20 min, which can be steadily correlated to the phosphorylation status of the GST-CTD. The dissolution of droplets was also observed in microscopic images. These data nicely confirm previous observations that the Cdk7 kinase is still active in pol II CTD condensates and that the condensates dissolve upon phosphorylation of the CTD hepta-repeats (21).

In the following experiment, the same assay was performed in the presence of 5% 1,6-hexanediol. As expected, addition of hexanediol abrogates the dissolution of the GST-CTD_[52]_ condensates despite the presence of Cdk7/CycH and ATP (Fig. 4*D*, upper panel). Within the time course monitored, no clearance of pol II CTD droplets was observed in the photometric assay, which is in agreement with the corresponding SDS-PAGE analysis demonstrating no phosphorylation of the pol II CTD (Fig. 4*D*, lower panel). The remaining of the condensates was also seen by light microscopy. Taken together this indicates that impairment of kinase activity by 1,6-hexanediol is irrespective of a phase separating component in the system.

## Discussion

The concept of membraneless organelles that form by LLPS gains more and more attention in many different fields in cell biology. The aliphatic dialcohol 1,6-hexanediol is widely used as a tool compound in phase separation studies to illustrate the reversible character of LLPS *in vitro* and in cells (26). In the present study, we demonstrate that 1,6-hexanediol greatly impairs kinase and phosphatase activity in a dose-dependent manner. Importantly, these findings were observed kinome-wide and independent of the capability of proteins to undergo LLPS. Experiments addressing LLPS use concentrations of up to 10% 1,6-hexanediol (24, 36). Based on our data, concentrations in this range would result in nearly complete inactivation of the kinases and phosphatases.

Phosphorylation has been described as both a negative and a positive modulator of phase separation (29, 37, 38). Many intrinsically disordered regions are subject to extensive phosphorylation. For example, the FUS low complexity domain is heavily phosphorylated by DNA-dependent protein kinase, which impairs LLPS (39). The use of 1,6-hexanediol could thus impair the appearance and abundance of this important signaling mark. This holds already at concentrations of 1,6-hexanediol where the agent inhibits kinase activity while condensates are not yet dissolved (Fig. 4*D*). The effect of hexanediol is thus irrespective of the LLPS and could potentially counteract mechanistic transitions in condensates. Importantly, the formation of many LLPS events has been reported to rely on kinase activities. The Pelkmans laboratory demonstrated a central role for dual-specificity regulated kinase 3 (DYRK3) in the regulation of stress granules and membraneless organelles in mitosis (40, 41). The kinase activity of DYRK3 was important to dissolve stress granules and different LLPS compartments during mitosis. This might have contributed to the observation of 1,6-hexanediol-resistant stress granules (3, 31).

In thermal stability measurements, we found that 1,6-hexanediol destabilizes proteins. However, at a concentration of 5% 1,6-hexanediol, we could not detect a significant loss of the CDK–cyclin interaction in pull-downs, indicating that the overall integrity of the protein structure is preserved (Fig. 3). We assume that reduced kinase and phosphatase activity is a consequence of small structural changes by the formation of bulk hydrogen bonds at the surface and interference with weak hydrophobic interactions with the substrate. As an example for the stability of a protein with regard to this dialcohol, ribonuclease A (13.7 kDa) has been successfully crystallized with a preserved three-dimensional structure in 70% hexanediol (42). The structure reveals the coordination of three 1,6-hexanediol molecules at the surface that interact through hydrogen bonding of the terminal alcoholic groups and hydrophobic interactions of the carbon chain. Such unspecific coverage of a proteins’ surface might impair enzymatic activity, possibly due to reduced substrate recognition ability. The effect of kinase inactivation by 1,6-hexanediol however appeared to be reversible upon dilution of the dialcohol after prolonged incubation times (Fig. 1*E*). This observation is in agreement with the high solubility of hexanediol in aqueous buffers and the reversible character when dissolving droplets.

In contrast to kinases and the phosphatase SSU72, the activity of the endonuclease DNase I was not affected by up to 5% 1,6-hexanediol, albeit the alcohol reduced the thermal stability of DNase I (Fig. 2*B* and 3*A*). Likewise, the DNA polymerases Q5 and OneTaq were differentially affected by hexanediol, losing their activity at either 10 or 2.5% of the chemical (Fig. 2*D*). This underlines that the effect of 1,6-hexanediol is not universal and has to be determined on a case-to-case basis. We found that at least 7.5% 1,6-hexanediol is required to dissolve phase-separated GST-CTD_[52]_ *in vitro* (Fig. 4*B*). Given the multitude of factors underlying phase separation, the concentration required to dissolve LLPS probably varies with respect to protein and experimental condition. Hence, the optimal 1,6-hexanediol concentration should be carefully evaluated. Two studies investigated long-term exposure of yeast and human cell cultures to 1,6-hexanediol and reported the formation of 1,6-hexanediol-resistant spots and increased cell death upon long-term treatment (3, 31). The authors argued for a careful use of the agent in cellular assays. Our study corroborates these findings by showing a direct impairment of kinase and phosphatase activity *in vitro*.

The Young lab recently reported that phase separation alters the pharmacologic potency of several cancer drugs by trapping them in phase-separated droplets and hence impairing target efficacy (17). Kinase inhibitors comprise a large class of anticancer agents and THZ-1, a covalent Cdk7 inhibitor, was indeed shown to accumulate in phase-separated condensates in the absence of its kinase target (17). Based on our findings, 1,6-hexanediol would be unsuited to analyze the pharmacokinetics and pharmacodynamics of kinase inhibitors when dissolving the condensates.

Besides the RNA pol II CTD, the transcription elongation kinase Cdk9 (P-TEFb) was shown to accumulate in condensates (21, 24). A recent study identified an intrinsically disordered region in the Cyclin T1 subunit to be responsible for P-TEFb mediated phase separation (24). P-TEFb mutants lacking the IDR of CycT1 were less active toward the phosphorylation of full-length human GST-CTD_[52]_ *in vitro* and were also less potent to drive transcription from a luciferase reporter gene. The hyperphosphorylation of the pol II CTD by P-TEFb was described to be promoted by the CycT1 IDR, which forms phase-separated droplets and/or speckles and recruits the pol II CTD into these compartments. However, the observed loss of hyperphosphorylation from intact P-TEFb when dissolving the phase-separated droplets by 1,6-hexanediol could be also an effect of reduced kinase activity when using this dialcohol.

1,6-Hexanediol has been used in a broad range of cellular assays to dissolve LLPS, as shown, *e.g.*, for G-bodies in yeast (43), chromosome pairing in *Schizosaccharomyces pombe* (44), viral infection (45), or DNA damage response (46). The transcriptional coactivators BRD4 and Med1 can form phase-separated droplets and treatment of cells with 1.5% 1,6-hexanediol for 30 min reduced BRD4 and Med1 occupancy at super-enhancers, accompanied by a loss of RNA pol II molecules at super-enhancer driven genes (20). Besides changes in LLPS, these effects could partially result from alterations in the phospho-proteom of the condensates. Likewise, a recent study reports that Epstein–Barr virus proteins EBNA2 and EBNALP control host gene expression through phase separation abilities on super-enhancers (47). Infected cells treated with 1% 1,6-hexanediol for 2 h showed reduced mRNA levels driven by super-enhancers, but not the control gene, which was accompanied by a loss of EBNA2 and EBNALP at super-enhancers. Intriguingly, transcription driven by super-enhancers is in particular vulnerable to Cdk7 kinase inhibition (48), which we find to be impaired already at low concentrations of 1,6-hexanediol.

Similar to transcription, heterochromatin domain formation was shown to underly phase separation processes. Two recent studies described the impact of heterochromatin protein 1 alpha (HP1α) on the regulation of heterochromatin formation by LLPS (36, 49). Probing *drosophila* and mammalian cell lines with 10% 1,6-hexanediol, a reduction of HP1α at heterochromatin was observed (36). Both studies show that HP1 phase separation is important for chromatin compaction and include mutational controls to address HP1α phase separation in chromatin compaction (36, 49). However, the ability of human HP1α to form condensates *in vitro* is critically dependent on its phosphorylation status (49). Hyperphosphorylation of the HP1α N-terminal extension results in an elongated shape, which allows the assembly of higher-order complexes. Importantly, HP1α-mediated LLPS sequesters known interaction partners such as Aurora B kinase into these condensates while excluding other proteins (49). Histone H3 phosphorylation at Ser10 by Aurora B during mitosis impairs HP1α binding resulting in chromatin decompaction (50). Chromatin compaction is thus positively and negatively regulated by phosphorylation.

With these examples discussed, we like to urge for a careful interpretation on the analysis of LLPS in cellular processes that involve phosphorylation reactions when using 1,6-hexanediol. Given the impact of this chemical on the enzymatic activity of kinases and phosphatases, changes in posttranslational modifications may contribute to the observed findings on condensate formation or dissolution.

## Experimental procedures

### Recombinant protein expression and purification

Recombinant proteins were expressed in *E. coli* BL21 DE3 pLysS bacterial cells (GST-CTD_[52]_, GST-CTD_[9]_, DYRK1A, SSU72) or in baculo virus infected *Sf9* insect cells (Cdk7/CycH, Cdk12/CycK, Cdk9/CycT1 f.l., Cdk9/CycT1 (1–272)).

Full-length human RNA pol II CTD was expressed with an N-terminal GST-tag for 16 h at 18 °C after induction by 0.1 mM IPTG at an OD_600_ of 0.8. For purification, cells were lysed in lysis buffer (50 mM HEPES pH 7.6, 150 mM NaCl, 5 mM β-mercaptoethanol) by sonication. The lysate was cleared from cell debris by centrifugation at 20,000 rpm in a JA25.50 rotor (Beckman-Coulter) and filtration through a PE filter with 0.45 μm pore size. GST-CTD_[52]_ was affinity purified using GSTrap columns (GE Healthcare). After elution in lysis buffer containing 10 mM GSH, the sample was concentrated and subjected to size-exclusion chromatography on a Superdex S200 pg column equilibrated in 50 mM HEPES (pH 7.6), 150 mM NaCl, 10% glycerol, 5 mM β-mercaptoethanol. GST-CTD_[9]_ was expressed for 4 h at 30 °C after induction with 0.5 mM IPTG at an OD_600_ of 0.6 to 1.2. GST-CTD_[9]_ was essentially purified as described above for full-length GST-CTD_[52]_ with the exception that gel filtration was performed with a Superdex S75 column.

Full-length human P-TEFb was expressed in *Sf9* cells for 72 h as His-Cdk9/GST-CycT1 from a pACEBac1-pIDK fusion plasmid using the MultiBac^Turbo^ system (51). Truncated P-TEFb was reconstituted from His-Cdk9 expressed in *Sf9* cells and GST-CycT1 (1–272) expressed in *E. coli* prior to affinity purification as described (52). Human Cdk12/CycK was coexpressed with CAK1 from *S. cerevisiae* and purified as described (53). Human GST-Cdk7 (2–346)/Cyclin H (1–323) was coexpressed in *Sf9* insect cells as described (54). A plasmid of human DYRK1A for bacterial expression in a pNIC28-Bsa4 vector was purchased from AddGene (Plasmid #38913). DYRK1A was expressed and purified as described (55).

SSU72 was expressed with an N-terminal, TEV-protease cleavable hexa-histidine-tag. *E. coli* cells were grown to OD_600_ of 0.7 to 1. Expression was induced with 0.4 mM IPTG for 16 h at 16 °C. For purification, cells were harvested and lysed in PBS containing 5 mM β-mercaptoethanol and 20 mM imidazole, pH 7.5. The lysate was cleared from cell debris by centrifugation at 15,000 rpm in a JA25.50 rotor (Beckman-Coulter) and filtration through a PE filter with 0.45 μm pore size. The lysate was applied to a 5 ml HisTrap FF crude column (GE Healthcare) using an ÄKTA prime FPLC system (GE Healthcare). After extensive washing with lysis buffer, His-SSU72 was eluted in Lysis buffer containing 250 mM imidazol. The His-tag was removed by TEV protease digest o/n at 4 °C and the sample further purified by size-exclusion chromatography on a Superdex S75 (16/600) pg column (GE Healthcare) equilibrated in 20 mM Tris-HCl (pH 7.6), 200 mM NaCl, 5% glycerol, 10 mM β-mercaptoethanol.

### Chemicals

1,6-Hexanediol at a purity of 99% was purchased from Sigma-Aldrich (#240117), heated to 45 °C, dissolved to 50% stock solution in water, and always mixed at RT in (v/v) percentages with protein samples.

### Kinase assays with recombinant protein

Radioactive kinase activity measurements were performed at a concentration of 0.2 μM kinase, 10 μM GST-CTD_[52]_ substrate, and 1 mM ATP containing 0.45 μCi [^32^P]/μl (Perkin Elmer) in kinase assay buffer (50 mM HEPES pH 7.6, 34 mM KCl, 7 mM MgCl_2_, 5 mM β-glycerophosphat, 2.5 mM DTE). Reactions were incubated for 15 min at 30 °C and stopped by adding EDTA to a final concentration of 50 mM. Reaction mixtures were spotted onto filter sheets of Amersham Protran nitrocellulose membrane (GE Healthcare). Filter sheets were washed three times for 5 min with PBS. Radioactivity was counted in a Beckman Liquid Scintillation Counter (Beckman-Coulter) for 1 min. Data were obtained from three independent experiments and normalized to a control without hexanediol treatment.

### Kinase assays with HeLa cell lysate

HeLa cells were lysed in lysis buffer (50 mM HEPES pH 7.6, 150 mM NaCl, 1% NP-40, 5 mM β-mercaptoethanol, 5 mM β-glycerophosphat) by gentle agitation for 15 min at 4 °C. Cell debris was removed by centrifugation at 15,000 rpm for 10 min in a benchtop centrifuge. For kinase assays, MgCl_2_ and ATP or ATPγS were added to a final concentration of 10 mM and 1 mM, respectively. Samples were incubated for 1 h at 30 °C. The reaction was stopped by addition of EDTA to a final concentration of 50 mM. Samples were alkylated with 2.5 mM PNBM for 30 min at room temperature. For analysis of the thio-phosphorylation by western blot, approximately 30 μg total protein was resolved on a 12% SDS-PAGE and blotted onto nitrocellulose. The blot was blocked in 5% milk powder in PBS containing 0.05% Tween20 (PBS-T) and incubated with the alkylation specific primary antibody (Abcam) diluted 1:5000 in PBS-T overnight and a secondary anti-rabbit IgG-HRP coupled antibody (Invitrogen, 1:10,000). Luminescence was detected in a BioRad ChemDocXRS+ system.

### Phosphatase assay

For phosphatase assays, GST-CTD_[9]_ was phosphorylated by incubation of 50 μM GST-CTD_[9]_ with 0.2 μM P-TEFb for 1 h at 30 °C in kinase assay buffer lacking β-glycerophosphate (50 mM HEPES pH 7.6, 34 mM KCl, 7 mM MgCl_2_, 2.5 mM DTE) in the presence of 2 mM ATP. For radiometric assays, ATP was supplemented with [^32^P]-γ-ATP. P-TEFb kinase activity was subsequently inhibited by the addition of flavopiridol to a final concentration of 1 μM.

For SSU72 phosphatase assays, highly phosphorylated GST-CTD_[9]_ (GST-pCTD_[9]_) was dephosphorylated using 5 μM SSU72 either in the presence or in the absence of 5% 1,6-hexanediol and incubated at 30 °C for the indicated times. Reactions were stopped by addition of 4xSDS-loading buffer and subjected to SDS-PAGE analysis or, in case of radiometric assays, phosphorylation was detected by liquid scintillation counting as described for kinase assays.

### DNase assay

Prior to DNase activity assays, the plasmid DNA was linearized by endonuclease digestion with *Eco*RI, followed by inactivation of the enzyme for 20 min at 65 °C, to allow a more accurate quantification of the DNA content, as nonlinearized plasmids exhibited different migration species due to supercoiled DNA. For DNase assay, 350 ng linearized plasmid DNA was digested with 1 μl DNase I (NEB, 2000 units/ml) diluted 1:10.000 in 1x DNase buffer (NEB) in a total reaction volume of 10 μl at 37 °C. The reaction was stopped at indicated time points by addition of 1 μl 50 mM EDTA and direct heat inactivation of the DNase at 70 °C for 10 min in a water bath.

DNA was analyzed by peqGreen (peqlab) staining in a 1% agarose gel. For quantification, bands were detected with a ChemDoc XRS+ system. The band intensities were quantified using the ImageLab5 software (BioRad).

### Polymerase assay

DNA polymerase activities were analyzed for two different enzymes in PCR amplification experiments. The Q5 high-fidelity DNA polymerase (M0491L) and the OneTaq quick-load DNA polymerase (M0509L) from New England BioLabs were used according to the manufacturers’ protocols. For the Q5 polymerase with proof-reading activity, PCR amplifications were performed on a DNA template with high GC content, as the encoded protein contains an elongated region of proline residues (56). To assure equal primer, DNA template, and enzyme concentrations, a master mix for six PCR reactions was prepared and aliquoted in separate tubes. As a last step, 1,6-hexanediol was added in increasing concentrations from 0 to 10%. After completion of the PCR experiment, the full reaction mix was subjected to gel electrophoresis. PCR products were analyzed by peqGreen (peqlab) staining in a 1% agarose gel.

### Thermal stability determination

Determination of thermal stability was performed by nano-differential scanning fluorimetry using a Prometheus device (NanoTemper). Cdk7/CycH and SSU72 were diluted to 5 μM in PBS. DNase I was diluted 1:4 in PBS and incubated with increasing concentrations of 1,6-hexanediol for 10 min prior to measurement. Thermal stability was monitored from 20 to 90 °C at a heating rate of 2 °C/min.

### Pull-downs

Ten micrograms of GST-Cdk7/CycH or His-Cdk9/GST-CycT1 (1–272) protein complexes was immobilized on GSH 4B sepharose beads in a total volume of 100 μl PBS. For input control, beads were collected immediately by centrifugation and CDK–cyclin complexes eluted with SDS-sample buffer. The other samples were incubated in PBS or in PBS with 5% 1,6-hexanediol for 1 h. Beads were collected by centrifugation and washed three times in PBS or PBS-hexanediol. CDK–cyclin complexes were eluted with SDS-sample buffer and subjected to Coomassie stained SDS-PAGE analysis.

### Pol II CTD phase-separation assay

To induce phase separation, GST-CTD_[52]_ was diluted to 10 μM in PBS containing 16% dextran. Phase-separated CTD was incubated with either H_2_O (control) or increasing concentrations of 1,6-hexanediol. Phase separation was monitored by measuring the turbidity of the sample at OD_600_ nm using a NanoDrop 2000c spectrophotometer (ThermoFisher Scientific).

## Data availability

All data of this study are contained within the article.

## Conflict of interest

The authors declare that they have no conflicts of interest with the contents of this article.
